# Influence of the agrochemicals used for rice and vegetable cultivation on insecticide resistance in malaria vectors in southern Côte d’Ivoire

**DOI:** 10.1186/s12936-016-1481-5

**Published:** 2016-08-24

**Authors:** Mouhamadou S. Chouaïbou, Behi K. Fodjo, Gilbert Fokou, Ouattara F. Allassane, Benjamin G. Koudou, Jean-Philippe David, Christophe Antonio-Nkondjio, Hilary Ranson, Bassirou Bonfoh

**Affiliations:** 1 Centre Suisse de Recherches Scientifiques en Côte d’Ivoire, 01BP 1303 Abidjan 01, Abidjan, Côte d’Ivoire; 2 Université Nangui Abrogoua, Abidjan, Côte d’Ivoire; 3 Liverpool School of Tropical Medicine, Pembroke Place, Liverpool, L3 5QA UK; 4 Laboratoire d’Ecologie Alpine (LECA), UMR 5553 CNRS-Université Grenoble-Alpes, BP 53, 2233 Rue de La Piscine, Bât. D Biologie, 38041 Grenoble, France; 5 Organisation de Coordination Pour La Lutte Contre Les Endémies En Afrique Centrale (OCEAC), PO Box 288, Yaoundé, Cameroon

## Abstract

**Background:**

Vector control can contribute to the development of resistance to insecticides in malaria vectors. As the swamps and wetlands used for some agricultural activities constitute productive breeding sites for many mosquito species, agricultural pest control may increase the selection pressure for insecticide resistance in mosquitoes. Understanding the use of agrochemicals by farmers is important to plan and initiate effective integrated pest and vector management interventions.

**Methods:**

A knowledge–attitude–practice study, using questionnaires, was undertaken with 102 rice farmers in Tiassalé and 106 vegetable farmers in Dabou (South Côte d’Ivoire) in order to generate information on pesticide usage. In addition, insecticide susceptibility bioassays were conducted using adult mosquitoes obtained from larvae collected within farms, and the persistence of agricultural pesticides in the farming environment, including sediment and mosquito breeding site water, was investigated by HPLC.

**Results:**

Herbicides and insecticides appeared to be the most frequently used pesticides for both crops. Amino phosphonates (mostly glyphosate) represented the most used herbicides (45 % for rice up to 89 % for vegetables). Pyrethroids appeared to be the most used insecticides (accounting for 90 % of all the insecticide use reported). Approximately 75 % of respondents had not been to school and do not understand product labels. Only about 45 % of farmers respect the recommended pesticide dosage and about 10–15 % of pesticides used for rice and vegetable, respectively, are not recommended for these crops. As per WHO criteria, the mosquito local populations from the two localities were resistant to three of the four insecticides tested, as mortalities were less than 35 % for deltamethrin, DDT and bendiocarb. Higher susceptibility was observed for malathion, although the population was considered resistant in Dabou (80 % mortality) and susceptible in Tiassalé (98 % mortality). With the exception of glyphosate, residues from each of six chemicals tested for were detected in each of the sites visited in the two localities.

**Conclusion:**

The study describes the use of insecticides and herbicides on crops and highlights the importance of considering agriculture practices when attempting to manage resistance in malaria vectors. Inter-sectoral collaboration between agriculture and public health is required to develop efficient integrated pest and vector management interventions.

**Electronic supplementary material:**

The online version of this article (doi:10.1186/s12936-016-1481-5) contains supplementary material, which is available to authorized users.

## Background

Vector control is a critical component of all malaria control strategies. The scale-up of two principal interventions, long-lasting insecticidal nets (LLINs) and indoor residual spraying (IRS), has been the major contributory factor to the 33 % reduction in malaria deaths in Africa in the last decade [1]. Unfortunately, the principal active ingredients used in these tools come from four classes of insecticide (pyrethroids, organochlorines (DDT), organophosphates, carbamates) to which malaria vectors are becoming increasingly resistant. This threat has led to the launch of the Global Plan for Insecticide Resistance Management in malaria vectors (GPIRM) in 2012 [2]. The GPIRM consists of five activities (described as five ‘pillars’) spanning the short, medium and long term, aiming to control insecticide resistance in order to ensure the continued effectiveness of current and future malaria vector control tools to prevent malaria transmission, morbidity and mortality. Vector control can contribute to the development of resistance, but the use of agricultural pesticides may also contribute to select resistance to insecticides used for vector control [3–8]. Indeed, the population of Africa has more than doubled in the last 30 years [9], increasing the demand for food and intensification of agricultural production to achieve food security. According to the New Partnership for Africa’s Development (NEPAD) [9], 530 million of the total African population (48 %) rely on agriculture and this is expected to exceed 580 million by 2020. Agrochemical purchase has increased dramatically in recent years and has resulted in significant reduction in harvest losses [9]. Given that swamp and wetlands used for some agricultural activities also constitute productive breeding sites for many mosquito species, agricultural pest control may become a threat to malaria vector control by contributing to the selection of insecticide resistance [3–8]. Therefore, understanding the use of agrochemicals by farmers is important to accurately initiate integrated pest and vector management interventions that could minimize the impact of crop protection on the development of insecticide resistance in malaria vectors. In this context, the present study aimed at investigating the practical use of agrochemicals by both rice and vegetable farmers in two rural areas of southern Côte d’Ivoire where populations have small-size farms dependent on family labour, with no use of machinery. To achieve this, a knowledge–attitude–practice (KAP) study, using questionnaires, was undertaken with 102 rice farmers in Tiassalé and 106 vegetable farmers in Dabou in order to generate information on pesticide usage, including nature of products, dosages, and frequency of treatments. Alongside the questionnaires, insecticide susceptibility bioassays were conducted using adult mosquitoes reared from larvae collected in breeding habitats found within farms. These samples were used to generate information on the frequency of resistant individuals in the population. Finally, the persistence of agricultural pesticides in the farming environment, including sediment and mosquito breeding site water, was investigated by high performance liquid chromatography (HPLC). Analysing all these data will enable a better understanding of the impact of agriculture on vector control and will guide insecticide resistance management strategies.

## Methods

### Study sites

The study was conducted between June and August 2015 in the localities of Tiassalé (5°53′54″N and 4°49′42″W) and Dabou (5°19′32″N and 4°22′36″W), located in the south of Côte d’Ivoire, respectively 100 km northwest and 30 km west of Abidjan, the economic capital. The climate is tropical and characterized by four seasons. A long rainy season (March–July) during which falls two-thirds of the annual rainfall, a short dry season (July–August), a short rainy season (September–November) and a long dry season (December–March). Tiassalé is characterized by rice production whereas vegetables are mainly cultivated in Dabou. Both sites have extensive mosquito breeding sites, which persist throughout the year [10]. The principal malaria vector is *Anopheles coluzzi* and *Anopheles gambiae* in a lower extent. Despite the role of *Anopheles funestus* in malaria transmission in Côte d’Ivoire, this species is not found in these locations. Malaria is the leading cause of morbidity among the population [11] with an incidence rate of 11.3 % in Tiassalé [12].

### Knowledge, attitude and practice (KAP) survey

Medical entomologists and social scientists worked together to prepare the questionnaire, which was piloted on a few farmers in order to assess their ease of comprehension. Questions were prepared to test ‘knowledge, attitude and practices’ related to pesticide use in the two study areas. Questions included in the ‘knowledge’ section were open-ended questions, without multiple-choice answers provided as this can result in guessing and therefore give a false impression of the knowledge of the farmers. Only farmers who gave their informed consent were included in the study. They were asked about their knowledge of the different pesticides used, including insecticides, fungicides, herbicides, fertilizers, and which chemical was used for which crop. Illiterate farmers were helped by one of the authors on behalf of the farmers. Questions included in the ‘attitude’ section were designed to gauge the prevailing attitudes, beliefs and understanding about the use of these agrochemicals. This section included questions on how farmers decide on which chemical to use, how they dilute these and how they proceed when they have the feeling that the product is not efficient. Finally, questions included in the ‘practice’ section were open-ended, designed to assess the practices of the respondents with regard to agrochemicals and focused on the dosage of pesticides and the frequency of treatments.

### Susceptibility bioassays

Mosquitoes were collected twice per week between May and July 2015 within the rice and vegetable furrows in the localities of Dabou and Tiassalé. In each locality, mosquitoes were collected at the larval stage from about 80–100 breeding sites. *Culex* and other larvae were discarded and only *Anopheles* larvae were pooled together according to their development stage, and reared using powdered TetraFin^®^ fish food. They were reared to adults under insectary conditions of 25–27 °C and 70–80 % relative humidity. Non-blood-fed, adult, female *A. gambiae s.l.* (the only *Anopheles* mosquito found) at 2–4 days post-emergence were tested using standard WHO susceptibility test kits [13] with insecticides belonging to the four classes used in public health, namely DDT (organochlorine), deltamethrin (pyrethroid), bendiocarb (carbamate), and malathion (organophosphate). Tests consisted of 1 h exposure to filter papers impregnated with each insecticide following the standard WHO procedure for adult mosquitoes at 25–27 °C and relative humidity of 70–90 % [13]. Each bioassay was performed with six batches of 20–25 females: four batches were exposed to impregnated filter papers and two non-exposed batches served as a control. The number of mosquitoes knocked down was recorded at 10 min intervals during the 1 h exposure period and the mortality was determined 24 h post-exposure. Following exposure, mosquitoes were supplied with 10 % honey solution and kept overnight under laboratory conditions prior to noting the 24 h mortality rates.

### Determination of pesticide residues in mosquito breeding water and soil samples

In both locations, larval soil and water were collected from three different larval sites and analysed for the presence of pesticide residues. Sampling was done in August 2015 during non-cultivation periods in order to avoid the impact of any recent treatment. According to the different agrochemical groups used by farmers, the six following compounds were tested: deltamethrin (pyrethroid, insecticide), chlorpyrifos ethyl (organophosphate insecticide), carbofuran (carbamate, insecticide), carbendazim (fungicide), glyphosate, and atrazine (herbicides). Three replicates of 50 g of soil samples were dried and ground while three replicates of 50–500 ml of breeding site water samples were simply decanted prior to pesticide extraction. Solid phase extraction (SPE) [14] was used for separation, purification and pre-concentration of pesticide residues before their detection by HPLC. Briefly, the SPE solid sorbent was conditioned using a solvent followed by the percolation of the sample to analyse through it. The next step consisted of the washing of the solid sorbent with solvent having low elution strength, to eliminate matrix components retained by the solid sorbent. The elution of the pesticide residues of interest was the obtained using appropriate solvent. Subsequently, the extracted compounds were loaded in HPLC system for separation and detection.

### Statistical analysis

The data were entered in a database created on the Epi Info software [15]. The KAP survey data were analysed using the Student *t* test through WassarStat software [16]. Statistically significant differences were considered when P value <0.05.

Susceptibility test data were interpreted based on WHO criteria [13], stating that mortality less than 90 % is indicative of resistance, mortality comprised between 90 and 97 % is suggestive of probable resistance and needs further investigation and mortality equal or more than 98 % is indicative of susceptibility. The mortality of test sample was calculated by summing the number of dead mosquitoes across all four exposure replicates and expressing this as a percentage of the total number of exposed mosquitoes. Abbott formula [17] was used to correct test mortality if mortality in the control was over 20 %.

Regarding the quantification of pesticides, the limit of detection (LOD) and limit of quantification (LOQ), determined as described by Keith et al. [18], were used to define the smallest concentration of any residues that could be reliably measured. Pesticide residues were defined in term of milligrams per litre of breeding site water or milligrams per kilogram of soil sediment. Values higher than the LOQ were considered and scored.

## Results

### KAP survey

#### Description of the group of farmers surveyed

A total of 208 farmers participated to the study (including 102 and 106 rice and vegetable cultivators, respectively) and all responses were entirely those of the farmers. Among the 102 rice growers, 44 (42.7 %) were females and 59 (57.3 %) were males. In Dabou, 38 (35.8 %) of the vegetable farmers were females and 68 (64.2 %) male. Ages ranged between 18 and 55 years and approximately 75 % of these farmers had not been to school; while 10 % had a primary school level and 15 % have been once in secondary school. About 90 % of respondents had more than 5 years of experience in the use of pesticides. Two types of rice crops were encountered, rain-fed crops with an average of one to two cycles in the year, and irrigated crops that could be made up to three times a year. Up to three cycles of vegetable cultivations could be found each year including lettuce, cabbage, spinach, cucumber, chili pepper, and pepper. The results presented here are for a single growing cycle.

#### Pesticides used for rice cultivation

**Fig. 1:**
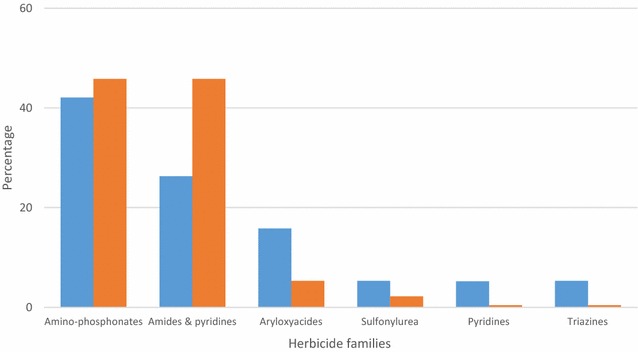
Herbicides used for rice cultivation in the locality of Tiassalé, South Côte d’Ivoire. The *blue bars* represent the proportion of each family of herbicide according to the total number of herbicides reported. The *red bars* represent the number of times that each family of herbicide was reported used by farmers according to the total number of herbicides reported. This corresponds to the rate of use

**Fig. 2:**
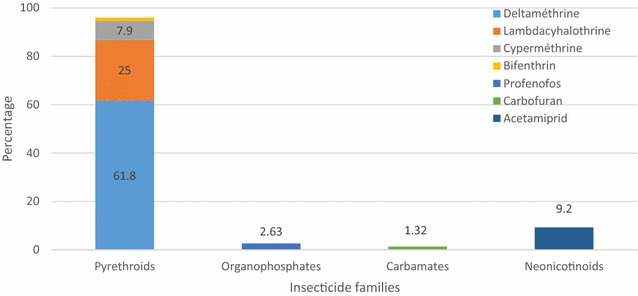
Insecticides used for rice cultivation in the locality of Tiassalé, South Côte d’Ivoire. Each *bar* represents the proportion of insecticide family reported by farmers according to the total number of insecticides reported. Among the pyrethroid family, each *colour* represents the proportion of pyrethroid-active ingredient according to the total number of insecticides reported to have been used by the farmers

Amino phosphonates (of which the only product used was glyphosate) was the chemical class most commonly reported (48 %) and appeared to be used by 46 % of farmers. This was followed by the amide and pyridines family (26.3 %), also used by about 46 % of farmers (Fig. 1). The family of aryloxyacides constituted 15.8 % herbicides reported and was used by 5.3 % of farmers. Herbicide families such as triazines, pyridines and sulfonylureas were poorly represented. Regarding insecticides (Fig. 2), pyrethroids were most commonly represented (92.11 %), alone or in combination with acetamiprid (neonicotinoid) (9.2 %) while other insecticide families, including organophosphates, organochlorines and carbamates accounted together for only 8 % of insecticides inventoried for rice cultivation. The utilization rate of pyrethroids was 11.6 times higher than for all other insecticides combined (P < 0.0001) (Fig. 2).

#### Pesticides used for vegetable cultivation

**Fig. 3:**
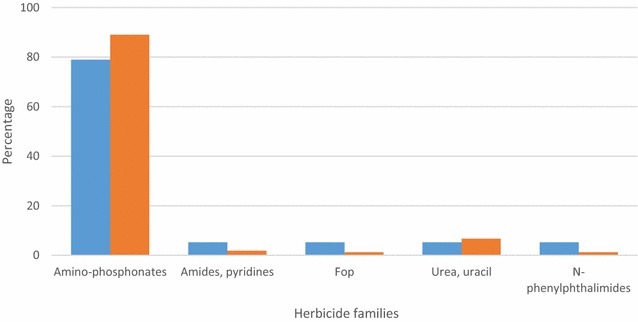
Herbicides used for vegetable cultivation in the locality of Dabou, South Côte d’Ivoire. The *blue bars* represent the proportion of each family of herbicide according to the total number of herbicides reported. The *red bars* represent the number of times that each family of herbicide was reported used by farmers according to the total number of herbicides reported. This corresponds to the rate of use

**Fig. 4:**
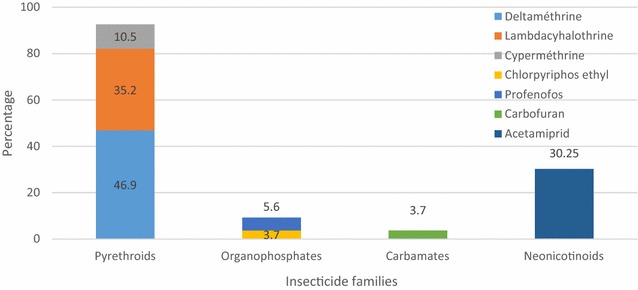
Insecticides used for vegetable cultivation in the locality of Dabou, South Côte d’Ivoire. Each *bar* represents the proportion of insecticide family reported by farmers according to the total number of insecticides reported. Among the pyrethroid family, each *colour* represents the proportion of pyrethroid-active ingredient according to the total number of insecticides reported to have been used by the farmers

In total, 33 different pesticides were identified (herbicides: 55.9 %, insecticides: 32.4 %, fungicides: 11.8 %) (Table 1). Insecticides (47.4 %) and herbicides (48.2 %) appeared to be the most frequently used pesticides. Amino phosphonates (glyphosate) represented 79 % of herbicide family used with 89 % of utilization frequency (Fig. 3). As for rice cultivation, pyrethroids appeared to be the most used insecticides (Fig. 4). They accounted for 72.7 % of all insecticides found, with an utilization rate of 94.9 %. They were essentially composed by lambdacyhalothrin and cypermethrin alone or in combination to the neonicotinoid acetamiprid. Organophosphates and carbamates represented, respectively, 18.2 and 9.1 % of insecticides identified, however, they were only weakly used with 3.9 and 0.6 % of utilization rate, respectively, 21-fold less than pyrethroids (P < 0.0001). Neonicotinoid in combination to pyrethroids accounted for 30.2 % of using rate according to all insecticides reported. This was three-fold higher compared to its using rate for rice cultivation (P < 0.01).

**Table 1:** Identification and status of different pesticides used for rice and vegetable cultivation in South Côte d’Ivoire

Locality	Name of product	Type	Active ingredient	Content	Family	Recom. status Y/N	Autho. status Y/N
Rice, Tiassalé	ADJOUMA SUPER	Herbicides	Glyphosate acid	480 g/l	Amino-phosphonates	Y	Y
	BIBANA	Herbicides	Glyphosate	360 g/l	Amino-phosphonates	Y	Y
	DRAGONHERBE	Herbicides	Glyphosate sel d’Isopropylammonium	41 %	Amino-phosphonates	Y	Y
	GLYPHADER	Herbicides	Glyphosate	360 g/l	Amino-phosphonates	Y	Y
	KALACH	Herbicides	Glyphosate	360 g/l	Amino-phosphonates	Y	Y
	KILLER	Herbicides	Glyphosate acid	360 g/l	Amino-phosphonates	Y	Y
	LADABA	Herbicides	Glyphosate	757 g/kg	Amino-phosphonates	Y	Y
	ROUND UP	Herbicides	Glyphosate	720 g/kg	Amino-phosphonates	Y	Y
	CALRIZ	Herbicides	Propanil, triclopyr	360 g/l, 72 g/l	Amide, pyridines	Y	N
	GARIL	Herbicides	Propanil, triclopyr	360 g/l, 72 g/l	Amide, pyridines	y	N
	MALORIL	Herbicides	Propanil, triclopyr	360 g/l, 72 g/l	Amide, pyridines	Y	N
	RICAL	Herbicides	Propanil, thiobencarb	200 g/l, 120 g/l	Amides, carbamates	Y	N
	SAKARIL	Herbicides	Propanil, triclopyr	360 g/l, 72 g/l	Amide, pyridines	Y	N
	CALLIHERBE	Herbicides	2,4-d Sel d’amine	720 g/l	Aryloxyacides	Y	Y
	DRAGONFLASH	Herbicides	2,4-d Sel de dimethylamine	720 g/l	Aryloxyacides	Y	Y
	HERBEXTRA	Herbicides	2,4-d Sel d’amine	720 g/l	Aryloxyacides	Y	Y
	VELPAR	Herbicides	Hexazinone	75 %	Triazines	Y	Y
	HERBO RIZ	Herbicides	Pyrazosulfuron-ethyl	100 g/kg	Sulfonylurates	Y	N
	GRAMOXONE	Herbicides	Paraquat	200 g/l	Pyridines	Y	N
	CYPERCAL	Insecticides	Cypermethrin	250 g/l	Pyrethroids	Y	Y
	DECIS	Insecticides	Deltamethrin	12,5 g/l	Pyrethroids	Y	Y
	KARATÉ	Insecticides	Lambdacyhalothrin	50 g/l	Pyrethroids	Y	Y
	K-OPTIMAL	Insecticides	Lambdacyhalothrin, acetamiprid	15 g/l, 20 g/l	Pyrethroids, neonicotinoids	Y	Y
	LAMBDAX	Insecticides	Lambdacyhalothrin	25 g/l	Pyrethroids	Y	Y
	CALFOS	Insecticides	Profenofos	375 g/l	Organophosphates	N	Y
	CALLI FAN SUPER	Insecticides	Bifenthrin, acetamiprid	20 g/l, 20 g/l	Pyrethroids, neonicotinoids	N	Y
	LAMBDAC	Insecticides	Lambdacyhalothrin, acetamiprid	30 g/l, 16 g/l	Pyrethroids, neonicotinoids	N	Y
	FURADAN	Insecticides	Carbofuran	5 %	Carbamates	Y	N
Vegetable, Dabou	BANKO PLUS	Fungicides	Chlorothalonil, carbendazim	550 g/l, 100 g/l	Organochlorines, carbamates	N	Y
	MANCOMAX	Fungicides	Mancozeb	800 g/kg	Carbamates	Y	Y
	RIMAFON	Fungicides	Dimethomorph, mancozeb	90 g/kg, 600 g/kg	Acid cinnamiques, carbamates	Y	Y
	ADJOUMA SUPER	Herbicides	Glyphosate acid	480 g/l	Amino-phosphonates	Y	Y
	BIBANA	Herbicides	Herbicides	360 g/l	Amino-phosphonates	Y	Y
	FANGA	Herbicides	Glyphosate	777 g/kg	Amino-phosphonates	Y	Y
	GLYPHADER	Herbicides	Glyphosate	360 g/l	Amino-phosphonates	Y	Y
	GRAMOXATE SUPER	Herbicides	Glyphosate	480 g/l	Amino-phosphonates	Y	Y
	HERBOUF	Herbicides	Glyphosate	360 g/l	Amino-phosphonates	Y	Y
	HERCULE	Herbicides	Glyphosate	480 g/l	Amino-phosphonates	Y	Y
	KALACH	Herbicides	Glyphosate	360 g/l	Amino-phosphonates	Y	Y
	KILLER	Herbicides	Glyphosate acid	360 g/l	Amino-phosphonates	Y	Y
	LADABA	Herbicides	Glyphosate	757 g/kg	Amino-phosphonates	Y	Y
	LAMACHETTE	Herbicides	Glyphosate	360 g/l	Amino-phosphonates	Y	N
	PUISSANCE	Herbicides	Glyphosate	780 g/kg	Amino-phosphonates	Y	Y
	ROUND UP	Herbicides	Glyphosate	720 g/kg	Amino-phosphonates	Y	Y
	TASMAN	Herbicides	Glyphosate sel d’ammonium	757 g/kg	Amino-phosphonates	Y	Y
	WURA SUPER	Herbicides	Glyphosate	480 g/l	Amino-phosphonates	Y	Y
	GARIL	Herbicides	Propanil, triclopyr	360 g/l, 72 g/l	Amide, pyridines	N	N
	IKOKADIGNE	Herbicides	Haloxyfop-R-methyl ester	108 g/l	Fop	N	Y
	SUPER	Herbicides	Bromacil, diuron	240 g/kg, 560 g/kg	Uracil, urea	N	Y
	TUSCANY	Herbicides	Flumioxazin	51 %	*N*-Phenylphthalimides	Y	N
	CYPERAX	Insecticides	Cypermethrin	50 g/l	Pyrethroids	Y	Y
	CYPERCAL	Insecticides	Cypermethrin	250 g/l	Pyrethroids	Y	Y
	DECIS	Insecticides	Deltamethrin	12.5 g/l	Pyrethroids	Y	Y
	LAMDOR	Insecticides	Lambdacyhalothrin	25 g/l	Pyrethroids	Y	Y
	KARATÉ	Insecticides	Lambdacyhalothrin	50 g/l	Pyrethroids	Y	Y
	K-OPTIMAL	Insecticides	Lambdacyhalothrin, acetamiprid	15 g/l, 20 g/l	Pyrethroids, neonicotinoids	Y	Y
	LAMBDAC	Insecticides	Lambdacyhalothrin, acetamiprid	30 g/l, 16 g/l	Pyrethroids, neonicotinoids	Y	Y
	POLYTHRINE	Insecticides	Cypermethrin, profenofos	30 g/l, 300 g/l	Pyrethroids, organophosphates	N	Y
	PYRICAL	Insecticides	Chorpyriphos-ethyl	480 g/l	Organophosphates	Y	Y
	PYRI FORCE	Insecticides	Chorpyriphos-ethyl	480 g/l	Organophosphates	N	Y
	FURADAN	Insecticides	Carbofuran	5 %	Carbamates	Y	N

*Recommended status* Yes/No (recommended by the manufacturer to be used or not for a crop), *Authorized status* Yes/No (Authorized by Ivorian legislation)

#### Status of pesticides and their use

Among the 19 herbicides inventoried for rice cultivation, only 12 (63.15 %) are licensed for use in Côte d’Ivoire, although all are suitable for use for rice cultivation according to the manufacturer guidance (Table 1). Only 14.7 % of farmers exclusively use authorized products (Table 2). Furthermore, pyridine and amides herbicides (26 % of herbicides, 45.8 % of rate of utilization) are prohibited by Ivorian regulations. As for insecticides, one carbamate (carbofuran) found in use in the current study is not authorized in Cote d’Ivoire. All pyrethroids used here are authorized for rice cultivation but other insecticide families are not (Table 1). Regarding vegetables, only four pesticides among the 33 are prohibited, however these four pesticides could reach 18 % of utilization rate. Up to 15 % of pesticides used are not recommended for vegetable cultivation by the manufacturers (Table 2). From the responses on the dosage per hectare of pesticides reported by the rice farmers, it was noticed that less than half (47.1 %) respected the recommended dose (Table 2). The same trends were seen for vegetable, with 43.4 % of farmers respecting the dose. Up to 75 % of rice farmers and 85 % of vegetable growers responded do not understand the labels. Up to 61 % of rice farmers and 90 % of vegetable farmers said they had never received any advice from a specialist. Furthermore, only 50 % of rice farmers and 60 % of vegetable farmers claimed to buy their products at authorized merchants (Table 2). Some farmers reported that they sometimes prepared their own chemical mixtures from different remaining chemicals prior to spraying.

**Table 2:** Use of pesticide for rice cultivation in Tiassalé, and vegetable in Dabou, South Côte d’Ivoire

	Locality of Tiassalé, ricefield, N = 102			Locality of Dabou, vegetable field, N = 106		
	Yes	Yes/no	No	Yes	Yes/no	No
Authorized pesticides by legislation encounted (%)	71.4	NA	28.6	87.9	NA	12.1
Use of authorized pesticides (%)	14.7	85.3	NA	82.1	17.9	NA
Recommended pesticides for the crop (%)	89.3	NA	10.7	84.8	NA	15.2
Respect of dosage (%)	47.1	52.9	NA	43.4	56.6	NA
Respect of dilution (%)	86.3	13.7	NA	55.7	44.3	NA
Understanding of labels (%)	24.5	NA	75.5	15.1	NA	84.9
Purchase in authorized shop (%)	59.8	NA	40.2	49.1	NA	50.9
Received advices from specialists (%)	39.2	NA	60.8	3.8	NA	96.2

*NA* not applicable

#### Insecticide susceptibility test

**Fig. 5:**
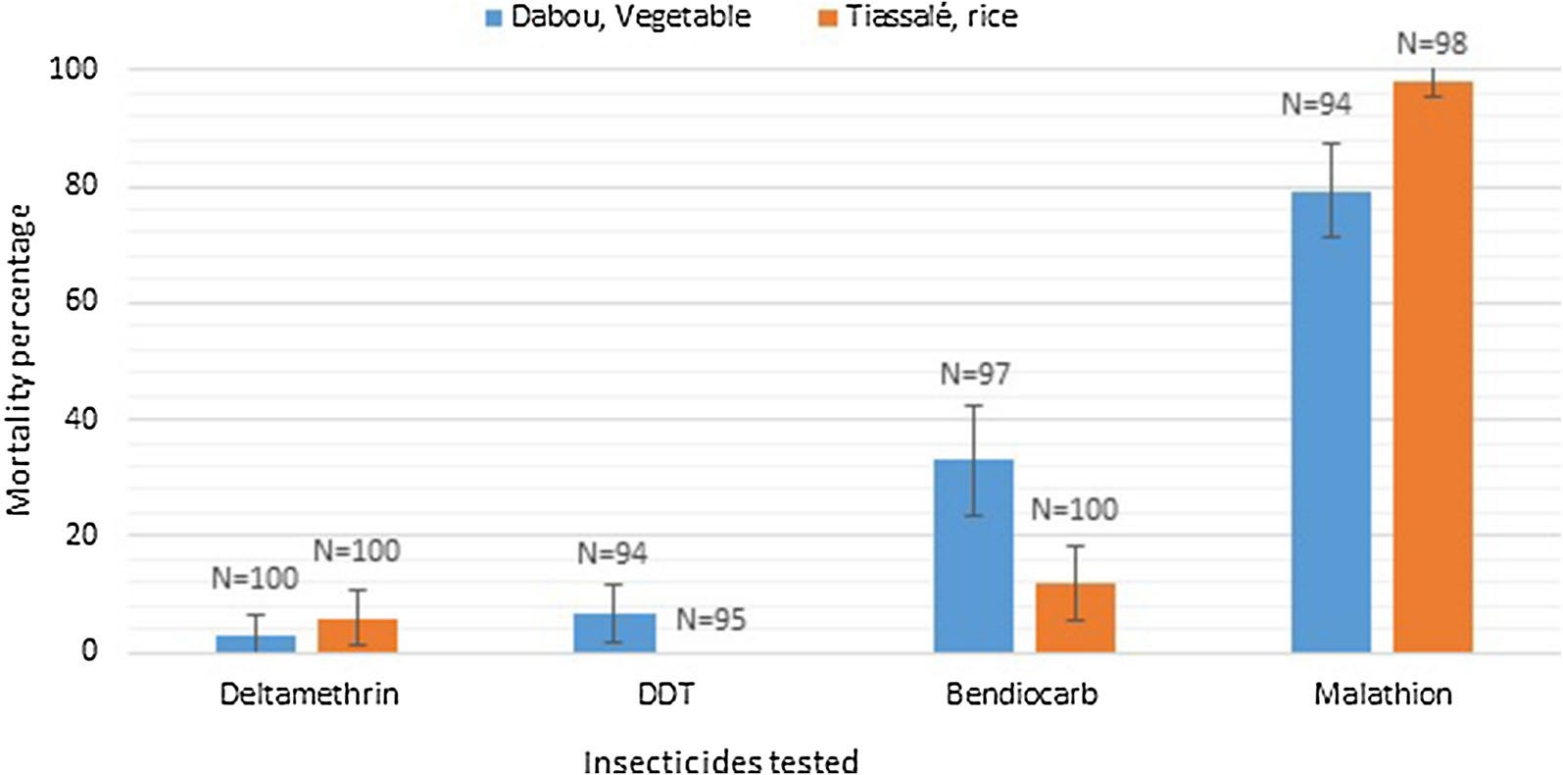
Mortality of *Anopheles gambiae s l* to four insecticides belonging to the four different classes of insecticides used in both public health and agriculture. The mortality rates were obtained after 1 h exposure of adult mosquitoes to insecticide-treated papers and 24 h holding period. Mosquitoes were collected at larval stage from furrows in rice and vegetable farms, respectively, in Tiassalé and Dabou. The *blue bars* represent the mortality rates obtained in the rice cultivation area and the *red bars* represent the mortality obtained in the vegetable cultivation area. The *error bars* represent the confidence intervals. The N on* top* of the *bars* represents the mosquito sample size

Mortalities in control groups (not exposed to insecticide) were consistently below 5 %, and no correction of mortality data was required. As per WHO criteria [13], the mosquito local populations were considered resistant to three of the four insecticides tested (Fig. 5) as mortalities were less than 35 % for deltamethrin, DDT and bendiocarb. The same trends were observed in the two localities. Higher susceptibility was observed for malathion, although the population was considered resistant in Dabou (80 % mortality) and almost susceptible in Tiassalé (98 % mortality).

#### Residues of pesticides in mosquito breeding site water and sediment

With the exception of glyphosate, residues for each of the six chemicals tested were found at levels higher than the quantification threshold limits in each of the sites visited in the two localities in at least one of the sample type (soil or breeding water) and sometimes in both. However, variations were observed between sampling sites from the same locality (Table 3). Deltamethrin residues were found in breeding site water in Tiassalé (0.03 mg/l = 1.5-fold LOQ) and in sediment both in Tiassalé (0.02 mg/kg = twofold LOQ) and Dabou (0.11 mg/kg = 11-fold LOQ). Chlorpyrifos ethyl was exclusively found in breeding site water with quantities comprised between 0.09 and 0.93 mg/l in Tiassalé (up to 45-fold LOQ), and 0.15–0.23 mg/l in Dabou (up to 11-fold LOQ). The carbamate carbofuran (which is prohibited) was found both in mosquito breeding site water (0.03 mg/l = 1.6-fold LOQ) and sediment in Dabou (0.04 mg/kg = 4.4-fold LOQ) and only in breeding site water in Tiassalé (0.02 mg/l = 1.1-fold LOQ). Of the herbicides, despite its high rate of use, glyphosate residues were not detected in Tiassalé or Dabou, whereas atrazine residues were present in both localities. This is despite no reported use of this chemical by farmers. Values in water from the breeding sites were 0.03 mg/l (1.6-fold LOQ) in Tiassalé and 0.72 mg/l (40-fold LOQ) in Dabou. In sediments, they were, respectively, 2.71 mg/kg (112-fold LOQ) and 2.31 mg/kg (96-fold LOQ). The fungicide carbendazim was also found present in the two localities. Residues in breeding site were found in Dabou only at 0.01 mg/l (1.25-fold LOQ). In ground sediment, values were 0.02 mg/kg in Tiassalé (1.8-fold LOQ) and 0.04 mg/kg in Dabou (3.6-fold LOQ).

**Table 3:** Residues of agrochemicals in both anopheles breeding sites’ water and soil sediments in rice cultivation area in Tiassalé, and vegetable area in Dabou, South Côte d’Ivoire

	Deltaméthrin (pyrethroids)		Chorpyriphos ethyl		Carbofuran (carbamate)		Glyphosate (herbicide)		Atrazine (herbicide)		Carbendazim (fungicide)	
	Breeding water	Soil sediment	Breeding water	Soil sediment	Breeding water	Soil sediment	Breeding water	Soil sediment	Breeding water	Soil sediment	Breeding water	Soil sediment
	mg/l	mg/kg	mg/l	mg/kg	mg/l	mg/kg	mg/l	mg/kg	mg/l	mg/kg	mg/l	mg/kg
Tiassalé 1	0.03 ± 0.002	0.02 ± 0.004	0.09 ± 0.004	ND	ND	ND	ND	ND	ND	ND	ND	ND
Tiassalé 2	ND	ND	ND	ND	ND	ND	ND	ND	0.03 ± 0.002	2.71 ± 0.04	ND	ND
Tiassalé 3	ND	ND	0.93 ± 0.03	ND	0.02 ± 0.006	ND	ND	ND	ND	ND	ND	0.02 ± 0.003
Dabou 1	ND	0.011 ± 0.02	0.17 ± 0.04	ND	0.03 ± 0.002	0.04 ± 0.002	ND	ND	0.72 ± 0.02	ND	0.01 ± 0.005	0.04 ± 0.005
Dabou 2	ND	ND	0.23 ± 0.05	ND	ND	ND	ND	ND	ND	2.31 ± 0.004	ND	ND
Dabou 3	ND	ND	0.15 ± 0.04	ND	ND	ND	ND	ND	ND	ND	ND	ND
LOD	0.008	0.007	0.007	0.005	0.006	0.003	0.005	0.012	0.006	0.008	0.005	0.002
LOQ	0.024	0.01	0.021	0.015	0.018	0.009	0.015	0.036	0.018	0.024	0.008	0.011

*LOD* limit of detection; *LOQ* limit of quantification; *ND* not detected

## Discussion

Agriculture is an essential activity in Tiassalé or Dabou for a large fraction of the population, and many farmers are illiterate as three-quarters of respondents have not been in school. The use of chemicals is the principal pest control strategy commonly applied by farmers to protect their investment. Unfortunately, these products, if not used correctly, can have direct environmental and health consequences and indirect consequences through the selection of insecticide resistance in malaria vectors. This constitutes a real threat for insecticide-based vector control and can lead to vector-control intervention failures, as reported in South Africa [19]. It appeared from the current study that there was a huge dependence on some specific pesticides. Herbicides from amino-phosphonates (glyphosates) and amide and pyridines (triclopyr, propanil) families, constituted the great majority of herbicides used. The same phenomenon is observed for pyrethroid insecticides (deltamethrin and lambdacyhalothrin), which constituted over 90 % of the insecticides used for both rice and vegetable. Some of these pyrethroid insecticides, such as K-optimal, lambdac or callifan-super, also contain acetamiprid, which is a neonicotinoid. This dependence may create a strong selective pressure in mosquito larvae breeding sites. A corollary can be observed for example, between the high dependence of farmers to pyrethroid insecticides and the high frequency of resistance to these insecticides observed in mosquitoes although pyrethroids are also mainly used in this area for vector control. In addition to the dependence on the same products (lambdacyhalothrin, cypermethrin, glyphosate), other factors contributing to the misusage of pesticides could be identified:

(i) The level of education: the great majority of the famers surveyed were illiterate and therefore could not read and understand labels, confirming what was observed by other authors [20, 21]. This limits the perceptions of users of environmental and health risks, but also the potential for written information on chemicals to be accurately used;
(ii) The pesticide efficacy in relation to dosage: applications did not closely match with manufacturers’ recommendations, meaning that understanding of pest biology, including susceptibility/resistance levels to chemicals used, is largely absent among workers. Thus, a potential development of resistance in crop pests is likely to be interpreted as a pesticide under dosing, which is followed by an increase in the dose of treatment;
(iii) Lack of guidance: although the surveyed farmers had in general more than 5 years of experience in the use of pesticides, this does not seem to have any impact on their way of proceeding with chemicals. Advice on the choice of a compound, the dilution rate and the dosage, the frequency of use are largely obtained from peers. This would probably have been different if farmers were receiving guidance from the Ministry of Agriculture or extension services, as over 90 % said to have never received any advice. An integrated pest management education programme in rice, onions and beans in Mali; tomato and cabbage in Senegal, contributed to increase yields up to 60 %, increased revenue and decreased pesticide use by 60–100 %; and,
(iv) The sales: the control of distribution and sale of pesticides is not effective. Multiple channels of supply seem to exist as only one to four authorized stores were found in the investigated localities, whereas many vendors were found selling their product alongside the market roads. This lack of effective regulation will likely lead to a repackaging and selling of obsolete or illegal stocks, as also noticed.

Furthermore, the use of chemicals with high persistence in environment is also to be considered. Indeed, quantification analysis revealed, among others, residues of atrazine although this herbicide has not been cited as in current use by farmers; this chemical has certainly been used in the past, which could explain its quantification, especially as this herbicide is known to have a very long persistence in soil and can last up to 1 year or more [22].

The use of the same insecticides both in agriculture and public health with the same target sites both in pests and in malaria vectors, could lead to much faster development of resistance to these compounds. Furthermore, the use of a varied range of xenobiotics (herbicides, fungicides, fertilizer, and other insecticides) could affect the metabolic system of mosquito larvae resulting in a broad spectrum of tolerance to several insecticides and select for resistance over generations [6–8, 23–29]. Atrazine has been shown to have an inducing role on enzymes responsible for insecticide degradation in mosquitoes [26]. Furthermore, selecting *A. gambiae* larvae with a mixture of agrochemicals increased their resistance to a broad range of insecticides at the adult stage [8].

Although the present study is not demonstrating a cause-effect relationship between the use of agrochemicals and development of resistance in malaria vectors, it further shows how these practices may favour the evolution of resistance as has been suggested by other studies [3, 4, 6–8, 30, 31]. Despite that, KAP investigations used here may show some limitations, such as accuracy in the answers. Empowering farmers with knowledge on agrochemical impact on health, environment and its potential role on malaria vector control, and enhancing their understanding on the safe handling, usage and management of pesticides is important to reduce the negative impact of agricultural chemicals, and will be of benefit for insecticide resistance management strategy in malaria vectors.

Current insecticide resistance management strategies recommend rotating insecticide classes used in IRS and/or using LLINs and IRS in combination [2]. These approaches assume that the use of insecticides in vector control is the major selective force. However, if a significant proportion of selection pressure is coming from agriculture for instance, then rotational use of different classes of insecticides in IRS may only have a limited efficacy. Efficient resistance management depends not only on information on the insect population (e.g., knowledge of vector susceptibility to insecticides, changing trends of resistance and their operational implications), but should also take into account the principal sources of insecticide resistance selection pressures. Hitherto, agricultural chemicals use in African regions was low. But today, given the substantial intensification of agriculture in the continent in recent years [9], followed by an important and uncontrolled use of pesticides for crop protection, the impact of agricultural chemicals on resistance development in malaria mosquitoes should not be underestimated. Thus, the spread of insecticide resistance is most likely a result of both vector control and the intensification of agriculture with associated pesticide inputs.

## Conclusion

Chemicals used in agriculture and public health constitute a double selection pressure of resistance in disease vectors. Inter-sectoral collaboration between agriculture and health is required to develop integrated pest and vector management interventions (IPVM). This process must also ensure that personnel, resources and structures are in place to manage the elimination of obsolete or retracted pesticides or hazardous chemicals and the strengthening of the capacity of farmers to use agrochemicals more effectively (Additional files 1, 2).

## Additional files

10.1186/s12936-016-1481-5 Questionnaire relative to chemical use for agriculture.
10.1186/s12936-016-1481-5 Chemical use for rice and vegetable cultivation in southern Côte d’Ivoire: crude answer’s from farmers.

## Abbreviations

HPLC: high performance liquid chromatography
WHO: World Health Organization
DDT: dichlorodiphenyltrichloroethane
LLIN: long-lasting insecticidal net
IRS: indoor residual spray
GPIRM: global plan for insecticide resistance management
NEPAD: New Partnership for Africa’s Development
KAP: knowledge–attitude–practice
SPE: solid phase extraction
LOD: limit of detection
LOQ: limit of quantification
IPVM: integrated pest and vector management
