# Supplementary material for: Influence of the agrochemicals used for rice and vegetable cultivation on insecticide resistance in malaria vectors in southern Côte d’Ivoire
Source: Malar J. 2016 Aug 24;15(1):426. doi: 10.1186/s12936-016-1481-5 (PMC4995742; doi:10.1186/s12936-016-1481-5)
Supplement: Supplementary file 1 — 10.1186/s12936-016-1481-5 Questionnaire relative to chemical use for agriculture. [file 12936_2016_1481_MOESM1_ESM.doc]

**Questionnaire sur l’utilisation des pesticides**

**AnoPest 2015**

| **N°** | **Questions** | **Modalités** | **Réponses** | **Sauts** |
| --- | --- | --- | --- | --- |
| Q101 | Localité | 1. Tiassalé  2. Autres (à préciser) | /__/ |  |
| Q102 | Sexe de l’enquêté | - - - 1. Masculin       2. Féminin | /__/ |  |
| Q103 | Age de l’enquêté | 1. [15-20 ans [  2. [20-25 ans [  3. [25-30 ans [  4. [30-35 ans [  5. [35-40 ans [  6. [40-45 ans [  7. [45-50 ans [  8. [50- 55 ans [  9. 55 ans et plus | /__/ |  |
| Q104 | Nationalité de l’enquêté | 1. Ivoirienne  2. Malienne  3. Burkinabé  4. Autres (à préciser) …………………… | /__/ |  |
| Q105 | Situation matrimoniale de l’enquêté | 1. Célibataire 2. Marié(e) monogame 3. Marié(e) polygame 4. Divorcé(e)/Séparé(e) 5. Veuf/veuve | /__/ |  |
| Q106 | Groupe ethnique de l’enquêté | 1. Mandé du Nord  2. Mandé du Sud  3. Akan  4. Gur  5. Krou | /__/ |  |
| Q107 | Niveau d’instruction de l’enquêté | 1. Aucun  2. Coranique  3. Primaire  4. Secondaire  5. Supérieur | /__/ |  |
| Q108 | Religion de l’enquêté | 1. Chrétienne 2.Musulmane  3. Animiste  4. Aucune  5. Autres (à préciser)…………………. | /__/ |  |
| Q109 | Depuis combien d’année utilisez-vous les pesticides ? | 1. < 1 an  2. [1-5 ans [  3. 5 ans et plus | /__/ |  |

| **N°** | **Questions** | | **Modalités** | **Réponses** | **Sauts** |
| --- | --- | --- | --- | --- | --- |
| Q110 | Etre-vous propriétaire de la plantation ? | | 1. Oui  2. Non | /__/ |  |
| Q111 | Qu’elle est l’activité de production | | 1. Riziculture  2. Maraichère  3. Autres (à préciser)……………… | /__/ |  |
| Q112 | Cette culture comprend combien de phases ? | | 1. Une phase  2. Deux phases  3. Trois phases | /__/ |  |
| Q113 | Quel groupe de pesticide est utilisé pour chaque phase ? | | 1. Insecticides  2. Herbicides  4. Fongicides  8. Acaricides  9. Engrais  16. Autres (à préciser)………………… | /__/ |  |
| Q114 | Pour chaque groupe de pesticide lister au moins 5 produits | | 1.  2.  3.  4.  5. | /__/ |  |
| Q115 | Nom commercial: | |  |  |  |
| Q116 | Le produit est-il du produit autorisé ? | | 1. Oui  2. Non | /__/ |  |
| Q117 | Famille: | |  |  |  |
| Q118 | Matière active: | |  |  |  |
| Q119 | Coût : | |  |  |  |
| Q120 | Dose appliquée (gramme ou litre/hectare) ? | |  |  |  |
| Q121 | La dose est-elle respectée ? | | 1. Oui  2. Non | /__/ |  |
| Q122 | Quelle est la dilution appliquée (gramme ou litre /1000 litres) ? | |  |  |  |
| Q123 | La dilution est-elle respectée ? | | 1. Oui  2. Non | /__/ |  |
| Q124 | Pendant quelles saisons de l’année  Le pesticide est-il utulisé ? | 1. Saison Pluvieuse  2. Saison sèche | | /__/ |  |
| Q125 | Fréquence ? | | 1. une fois  2. Deux fois  3. Trois fois | /__//__/ |  |
| Q126 | Nom commercial: | |  |  |  |
| Q127 | Le produit est-il du produit autorisé ? | | 1. Oui  2. Non | /__/ |  |
| Q128 | Famille: | |  |  |  |
| Q129 | Matière active: | |  |  |  |
| Q130 | Coût : | |  |  |  |
| Q131 | Dose appliquée (gramme ou litre/hectare) ? | |  |  |  |
| Q132 | la dose est-elle respectée ? | | 1. Oui  2. Non | /__/ |  |
| Q133 | Quelle est la dilution appliquée (gramme ou litre /1000 litres) ? | |  |  |  |
| Q134 | La dilution est-elle respectée ? | | 1. Oui  2. Non | /__/ |  |
| Q135 | Pendant quelles saisons de l’année  Le pesticide est-il utulisé ? | 1. Saison pluvieuse  2. Saison sèche | | /__/ |  |
| Q136 | Fréquence ? | | 1. Une fois  2. Deux fois  3. Trois fois | /__/ |  |
| Q137 | Nom commercial | |  |  |  |
| Q138 | Le produit est-il du produit autorisé ? | | 1. Oui  2. Non | /__/ |  |
| Q139 | Famille: | |  |  |  |
| Q140 | Matière active: | |  |  |  |
| Q141 | Coût : | |  |  |  |
| Q142 | Dose appliquée (gramme ou litre/hectare) ? | |  |  |  |
| Q143 | La dose est-elle respectée ? | | 1. Oui  2. Non | /__/ |  |
| Q144 | Quelle est la dilution appliquée (gramme ou litre /1000 litres) ? | |  |  |  |
| Q145 | La dilution est-elle respectée ? | | 1. Oui  2. Non | /__/ |  |
| Q146 | Pendant quelles saisons de l’année  Le pesticide est-il utulisé ? | 1. Saison Pluvieuse  2. Saison sèche | | /__/ |  |
| Q147 | Fréquence ? | | 1. une fois  2. Deux fois  3. Trois fois | /__/ |  |
| Q148 | Nom commercial: | |  |  |  |
| Q149 | Le produit est-il du produit autorisé ? | | 1. Oui  2. Non | /__/ |  |
| Q150 | Famille: | |  |  |  |
| Q151 | Matière active: | |  |  |  |
| Q152 | Coût : | |  |  |  |
| Q153 | Dose appliquée (gramme ou litre/hectare) ? | |  |  |  |
| Q154 | La dose est-elle respectée ? | | 1. Oui  2. Non | /__/ |  |
| Q155 | Quelle est la dilution appliquée (gramme ou litre /1000 litres) ? | |  |  |  |
| Q156 | La dilution est-elle respectée ? | | 1. Oui  2. Non | /__/ |  |
| Q157 | Pendant quelles saisons de l’année  Le pesticide est-il utulisé ? | 1. Saison Pluivieuse  2. Saison sèche | | /__/ |  |
| Q158 | Fréquence ? | | 1. une fois  2. Deux fois  3. Trois fois | /__/ |  |
| Q159 | Nom commercial | |  |  |  |
| Q160 | Le produit est-il du produit autorisé ? | | 1. Oui  2. Non | /__/ |  |
| Q161 | Famille: | |  |  |  |
| Q162 | Matière active: | |  |  |  |
| Q163 | Coût : | |  |  |  |
| Q164 | Dose appliquée (gramme ou litre/hectare) ? | |  |  |  |
| Q165 | La dose est-elle respectée ? | | 1. Oui  2. Non | /__/ |  |
| Q166 | Quelle est la dilution appliquée (gramme ou litre /1000 litres) ? | |  |  |  |
| Q167 | La dilution est-elle respectée ? | | 1. Oui  2. Non | /__/ |  |
| Q168 | Pendant quelles saisons de l’année  le pesticide est-il utulisé ? | 1. Saison Pluivieuse  2. Saison sèche | | /__/ |  |
| Q169 | Fréquence ? | | 1. une fois  2. Deux fois  3. Trois fois | /__/ |  |
| Q170 | Y a-t-il des étiquètes sur les contenants des pesticides que vous employez ? | | 1. Oui  2. Non | /__//__/ |  |
| Q171 | Si oui comprenez-vous les étiquètes ? | | 1. Oui  2. Non | /__/ |  |
| Q172 | Quel est le lieu d’approvisionnement des pesticides (Localité) ? | | 1. Abidjan  2. Tiassalé  4. Autres | /__/ |  |
| Q173 | Qui est ton fournisseur ? | | 1. Commerçant agrée  2. Particulier  4. Autres | /__/ |  |
| Q174 | Pour combien de traitement achetez-vous les pesticides ? | | 1. Un traitement  2. Plusieurs traitements | /__/ |  |
| Q175 | Les pesticides sont-ils stockés ? | | 1. Oui  2. Non | /__/ |  |
| Q176 | Si oui, où les stockez-vous ? | | 1. A la maison :  2. Au grenier :  3. Autres | /__/ |  |
| Q177 | les pesticides sont-ils utilisés pour autres choses ? | | 1. Traiter les maisons  2. La pelouse  4. Autres | /__/ |  |
| Q178 | Qui vous a conseillez l’utilisation de ces pesticides ? | | 1. ANADER  2. CNRA  3. Commerçant  4. Cultivateur  5. Autres | /__/ |  |
| Q179 | Sur quoi est basé votre critère de choix du pesticide ? | | 1. Le prix  2. L’efficacité  3. La disponibilité  4. Autres (Préciser) | /__/ |  |
| Q180 | Quel est votre type d’exploitation ? | 1. Individuelle  2. Familiale  3. Communautaire  4. Industrielle  5. Autres (à préciser) | | /__/ |  |
|  |  | |  |  |  |
